# Structure–activity relationships for the G-quadruplex-targeting experimental drug QN-302 and two analogues probed with comparative transcriptome profiling and molecular modeling

**DOI:** 10.1038/s41598-024-54080-2

**Published:** 2024-02-11

**Authors:** Ahmed Abdullah Ahmed, Shuang Chen, Maria Roman-Escorza, Richard Angell, Sally Oxenford, Matthew McConville, Naomi Barton, Mihiro Sunose, Dan Neidle, Shozeb Haider, Tariq Arshad, Stephen Neidle

**Affiliations:** 1 The School of Pharmacy, University College London, London, WC1N 1AX UK; 2 Now at Guy’s Cancer Centre, Guy’s Hospital, London, SE1 9RT UK; 3 Now at Medicines Discovery Institute, Cardiff University, Cardiff, CF10 3AT UK; 4 Now at Artios Ltd, Cambridge, CB22 3FH UK; 5 Sygnature Discovery Ltd, BioCity, Nottingham, NG1 1GR UK; 6 Tax Policy Associates, London, EC1R 0ET UK; 7 Qualigen Therapeutics Inc, Carlsbad, CA 92011 USA

**Keywords:** Gastrointestinal cancer, Medicinal chemistry, Target identification, Cancer, Chemical biology, Drug discovery, Oncology

## Abstract

The tetrasubstituted naphthalene diimide compound QN-302 binds to G-quadruplex (G4) DNA structures. It shows high potency in pancreatic ductal adenocarcinoma (PDAC) cells and inhibits the transcription of cancer-related genes in these cells and in PDAC animal models. It is currently in Phase 1a clinical evaluation as an anticancer drug. A study of structure–activity relationships of QN-302 and two related analogues (CM03 and SOP1247) is reported here. These have been probed using comparisons of transcriptional profiles from whole-genome RNA-seq analyses, together with molecular modelling and molecular dynamics simulations. Compounds CM03 and SOP1247 differ by the presence of a methoxy substituent in the latter: these two compounds have closely similar transcriptional profiles. Whereas QN-302 (with an additional benzyl-pyrrolidine group), although also showing down-regulatory effects in the same cancer-related pathways, has effects on distinct genes, for example in the hedgehog pathway. This distinctive pattern of genes affected by QN-302 is hypothesized to contribute to its superior potency compared to CM03 and SOP1247. Its enhanced ability to stabilize G4 structures has been attributed to its benzyl-pyrrolidine substituent fitting into and filling most of the space in a G4 groove compared to the hydrogen atom in CM03 or the methoxy group substituent in SOP1247.

## Introduction

G-quadruplexes (G4s) are higher-order structures that can be formed in DNA or RNA^1,2^ by the association of short guanine (G)-tracts such that two or more G-quartets are formed, which result in the folding of these sequences into four-stranded arrangements^2–4^, that are fundamentally distinct from duplex DNA or RNA. X-ray crystallographic and NMR studies have revealed the complexity of many G4 arrangements^5^. The existence of discrete G4-forming sequences in the human genome^6,7^ and their over-representation in the promoter and untranslated regions of many cancer-associated genes^8–14^, has led to an anticancer concept, of stabilizing G4s against unwinding with appropriate and selective small-molecule compounds, leading to inhibition of transcription, translation, or replication^15–19^, depending on the location of a G4 sequence within the targeted G4 genes. Several thousand low molecular weight compounds have been examined for G4 affinity and ability to target individual G4s, notably those in the promoters of the *MYC*, *KIT*, *KRAS* and *BCL2* genes (see for example, Refs.^20–25^).

**Figure 1:**
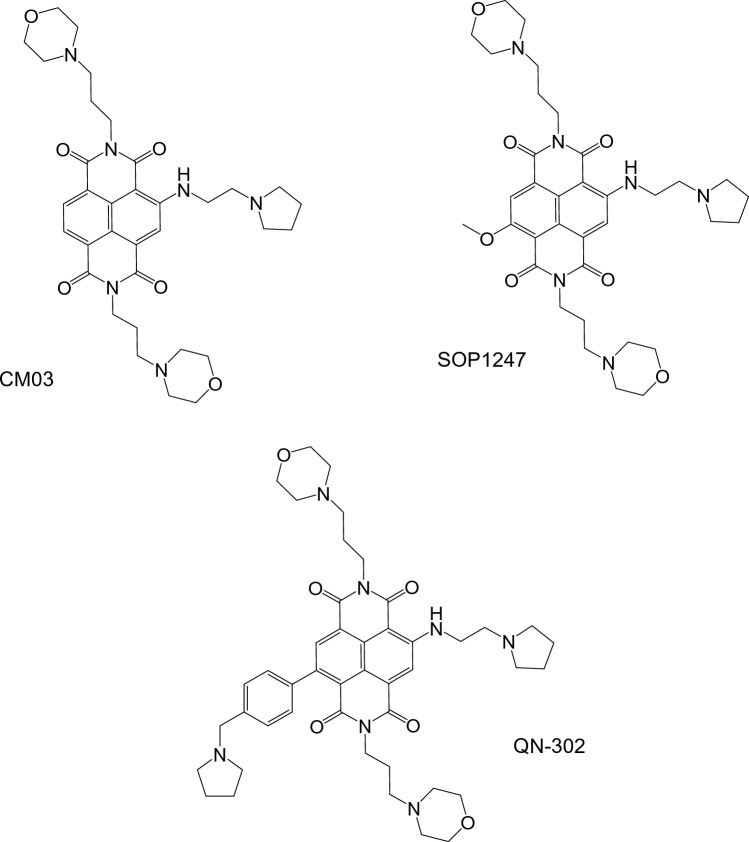
Molecular structures of the three compounds discussed here.

Compounds based on a naphthalene diimide core^26–32^ have received considerable attention in view of their demonstrated ability to exhibit anticancer activity. We have devised several generations of them, developed using structure-based design and medicinal chemistry/pharmacology to optimize hit compounds^33–39^. Lead compounds have shown high G4 affinity and cell growth inhibition ability with GI_50_ values typically < 1 µM in a panel of cancer cell lines. Compound CM03^40,41^ (Fig. 1a) with three side chains terminating in cationic charged end-groups, has a typical GI_50_ value of ca 10 nM in cancer cell lines and displays activity in in vivo models for pancreatic ductal adenocarcinoma (PDAC). A subsequent medicinal chemistry/pharmacology campaign to optimize the G4 affinity and biological activity of CM03 resulted in two further lead compounds, SOP1247 and QN-302 (Fig. 1b,c). These are related by changes in the substitution pattern at the 4th position on the naphthalene diimide core, while at the same time maintaining as identical all other substituents. QN-302^42^, with a benzyl-pyrrolidine group, has outstandingly potent cell growth inhibitory activity in PDAC cells, with GI_50_ values of ca 1–2 nM and corresponding superior potency compared to CM03 and SOP1247 (with a methoxy group replacing the benzyl-pyrrolidine) in several animal models of PDAC and other human cancers. QN-302 is also a potent binder to various G4 structures^42^. It was subsequently selected by Qualigen Therapeutics Inc as a clinical candidate and has recently been given IND clearance by the Food and Drug Administration in the USA to initiate Phase 1a clinical trials. The first patient enrolled for this trial was reported in early November 2023.

We report here on a comparative analysis of the transcriptional profile of all three compounds, CM03, SOP1247 and QN-302 in the MIA-PaCa2 PDAC cell line, using a RNA-seq approach. Transcriptome (RNA-seq) data has also been included from CM03 in a MIA-PaCa2 cell line generated by us using repeated passage to be gemcitabine-resistant^41^ and in a 2nd PDAC cell line, PANC-1^40^. Molecular modelling and molecular dynamics simulations have been used to rationalize differences in behavior between the three compounds, where a human telomeric G4-duplex structure has been taken as a paradigm for G4s embedded in duplex DNA.

## Results

### Cellular activity

The cellular potency of QN-302 in a small panel of PDAC cell lines is consistently ca tenfold higher than the two closely related G4 compounds SOP1247 and CM03 (Table 1). CM03 shows small but significantly superior potency compared to SOP1247 in three out of the four PDAC cell lines in the panel. This may be a consequence of the methoxy group in the latter compound, which would have a slight effect on hydrophobicity. It does not appear to affect the ability of SOP1247 compared with CM03 to stabilize a human telomeric G4 since they produce closely similar ΔT_m_ values. On the other hand, QN-302 has superior G4 stabilizing ability^43^, consistent with its enhanced cell growth inhibitory and in vivo potency^42^.

**Table 1:** Data for cell growth inhibition and G4 melting stabilization (from a FRET study with human telomeric (htel) G4).

	CM03	SOP1247	QN-302
GI_50_ 96 h (nM)			
MIA-PaCa2	9.0	13.8	1.3
PANC-1	15.6	15.7	1.4
CAPAN-1	26.5	38.8	5.9
Bx-PC3	15.5	20.5	2.6
MIA-PaCa2-GemR	14.9	N/A	3.8
ΔT_m_ (°)			
	17.6	18.4	23.1

Esds for the IC_50_ data are ± 0.5 nM, and for the ΔT_m_ data are ± 0.5°. Cell growth inhibition data for compounds CM03 and QN-302 in the parental cell lines have been previously published^40,42^, though re-measured for this study. MIA-PaCa2-GemR is a gemcitabine-resistant cell line^41^.

### Global expression changes

**Figure 2:**
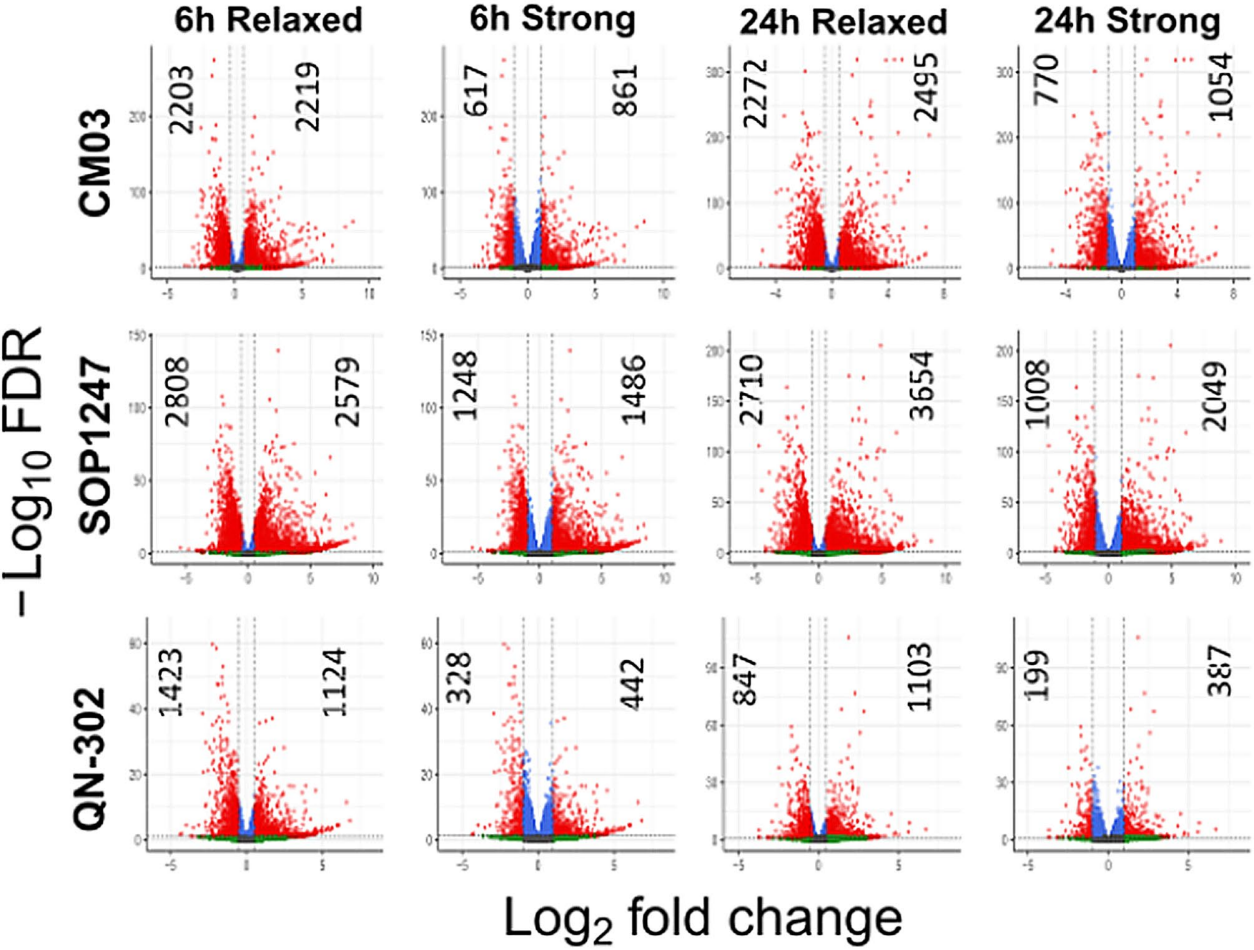
Volcano plots of Total numbers of differentially expressed genes induced by drug dosage in MIA-PaCa2 cells for the three G4-binding compounds (CM03, SOP1247 and QN-302) at two different time points (6 h and 24 h), from RNA-seq analyses. The differentially expressed genes were sorted into 4 subgroups based on the log_2_ fold change (log_2_FC) and false discovery rate (FDR): Down (Log_2_ FC ≤  − 0.5, FDR ≤ 0.1), Up (Log_2_ FC ≥ 0.5, FDR ≤ 0.1), Down strong (Log_2_ FC ≤  − 1.0, FDR ≤ 0.05) and Up strong (Log_2_ FC ≥ 1.0, FDR ≤ 0.05). The horizontal dotted lines indicate FDR cut-offs and the vertical dotted lines indicate Log_2_ FC cut-offs for (left) down-regulated and (right) up-regulated genes. The numbers on the plot indicate the number of DEGs that meet the cut-offs.

Figure 2 shows that QN-302 affects the down-and up-regulation of fewer genes than do the other two compounds, in accord with results from the more limited earlier studies^42^. At the 24 h time-point, the differences in numbers affected are at least threefold. Thus, QN-302 is a more selective agent than either of the other two compounds in MIA-PaCa2 cells and compared to CM03 in PANC-1 and MIA-PaCa2-gemcitabine-resistant cells.

Table 2 highlights a selection of the major downregulated pathways and genes for all three compounds in MIA-PaCa2 cells, as well as CM03 in gemcitabine-resistant MIA-PaCa2 cells and in the PANC-1 PDAC cell line. The Table shows that all the compounds induce changes in hedgehog, WNT/β-catenin, axon guidance, signal transduction and hippo pathways as well as in some transcriptional, chemokine and transporter genes. However, there are numerous differences in the responses found for individual genes. It is notable that the differences are most apparent for QN-302 compared to the other compounds as well as compared to CM03 in both PANC-1 and MIA-PaCa2 gemcitabine-resistant cell lines. Thus, in the *GLI* family (glioma-associated oncogenes) coding for zinc finger proteins in the hedgehog pathway, the *GLI4* gene is the dominant down-regulated of the four genes *GLI1-4* in all except the QN-302-treated cell line, where *GLI1* is the most down-regulated gene of the four. QN-302 has lesser effects on the listed constituents of the WNT and hippo pathways compared to the other compounds. The *NTN4* gene in the axon guidance pathway is highly down-regulated by QN-302, whereas it is upregulated by both CM03 and SOP1247. This gene is also involved in the WNT/β-catenin pathway. The *PRDM16* gene, which appears to act as a transcription factor and as a methyltransferase, is down-regulated by all compounds (Table 2), as is the transcriptional co-repressor gene *CBFA2T3*: both are notably less down-regulated by QN-302 than by the other compounds. The chemokine gene *CX3CL1*, on the other hand, is most strongly down-regulated by QN-302, as is the transporter gene *CLIC3*. The folate transporter gene *SLC19A1* is consistently highly down regulated by all three compounds and in the resistant and PANC-1 cells with CM03. Components of the RAS and P38 MAP kinase signal transduction pathways are all affected, though to a lesser extent than this transporter gene, with the *MAPK11* gene being consistently more affected than the other genes listed in Table 2. The *S100P* gene, which codes for the small calcium-binding protein S100P, is highly down-regulated by QN-302. The P value for this gene is on the edge of significance: however as previously reported^44^, changes at the transcriptional and translational level found in vivo for this gene in QN-302-treated MIA-PaCa2 xenograft tissue, are highly statistically significant, so this gene is retained in the list here.

**Table 2:** Selected down-regulated genes after 24 h drug exposure, with log_2_ FC values across all datasets, from RNA-seq analyses.

Functional relevance, gene name	SOP1247 in MIA- PaCa2	CM03 in MIA- PaCa2	CM03 in MIA- PaCa2-GemR	CM03 in PANC-1	QN-302 in MIA-PaCa2	PQs
Hedgehog pathway						
GLI1	− 0.13	0.03	− 0.57	− 0.57	− 1.84	15
GLI2	− 0.74	− 1.24	0.00	0.38	− 0.15	71
GLI3	2.63	− 0.58	0.00	0.28	− 0.47	13
GLI4	− 2.67	− 1.26	− 2.76	− 1.71	− 0.90	11
WNT pathway						
WNT5B	− 0.66	− 0.97	0.34	− 0.01	− 0.58	8
DVL1	− 1.87	− 1.35	− 2.68	− 1.56	− 0.77	30
AXIN1	− 0.92	− 0.72	− 1.35	− 0.74	− 0.39	18
APC2	− 3.20	− 1.61	− 3.17	− 1.62	− 1.06	4
Axon guidance						
PAK1	− 1.39	− 1.13	− 1.74	− 1.11	− 0.22	14
ROBO3	0.08	− 0.53	− 1.09	− 0.85	0.23	19
PLXNA1	− 1.62	− 1.56	− 2.55	− 1.50	− 0.92	54
NTN4	3.47	4.04	N/A	0.95	− 2.46	13
Hippo pathway						
TAZ	− 0.88	− 1.10	− 1.49	− 0.74	− 0.56	8
TEAD2	− 0.52	− 0.91	− 0.37	0.21	− 0.61	19
TEAD3	− 0.14	− 0.66	− 0.33	0.07	− 0.43	29
TEAD4	− 0.50	− 0.31	− 0.77	− 0.04	− 0.13	34
SHANK2	− 2.4	− 1.96	N/A	− 0.44	− 0.37	203
SHANK3	− 1.34	− 0.85	− 0.95	− 1.12	− 0.11	42
Transcriptional genes						
PRDM16	− 3.88	− 3.39	− 3.48	− 3.14	− 1.91	260
TP73	− 1.49	− 1.19	− 2.37	− 0.99	− 0.61	91
CBFA2T3	− 2.90	− 2.86	− 3.32	− 2.24	− 1.50	96
MYC	− 0.02	0.34	0.71	0.71	− 0.07	4
Chemokines						
CX3CL1	− 1.08	− 1.82	N/A	0.62	− 2.91	5
CXCL1	− 2.90	− 1.38	− 1.38	− 1.69	− 1.37	1
Signal transduction pathways						
KRAS	0.22	− 0.15	− 0.17	0.05	− 0.10	4
MAPK11	− 2.12	− 2.56	− 3.03	− 1.59	− 1.72	18
MAPK12	− 1.11	− 1.23	− 0.68	− 1.44	− 0.70	18
AKT1	− 1.20	− 0.98	− 1.55	− 1.04	− 0.37	44
ARF6	− 0.99	− 0.54	− 0.99	− 0.69	− 0.22	7
IQSEC1	− 0.79	− 0.53	− 0.80	− 0.50	− 0.20	82
Transporters						
SLC19A1	− 4.70	− 3.94	− 5.25	− 2.05	− 2.27	38
SLC29A1	− 0.49	− 0.16	− 0.60	− 0.15	− 0.17	25
CLIC3	1.05	0.92	N/A	− 0.29	− 2.77	6
Misc function						
HSPA1A	− 0.21	− 0.12	− 0.46	0.01	− 1.15	3
BCL2	− 1.76	− 1.24	− 1.82	N/A	− 0.48	21
hTERT	− 3.32	− 2.47	− 4.21	− 0.98	− 1.03	15
S100P	− 0.53	N/A	1.47	N/A	− 3.23	60
RTN4R	− 2.87	− 2.23	− 2.77	− 1.85	− 1.87	28
KRT16	− 4.06	−	−	0.56	− 1.98	2

PQs represent the estimated number of putative quadruplex-forming sequences, taken from^40–42^.

To identify the differences in gene responses that may be responsible for the increased potency of QN-302, unsupervised hierarchical clustering was performed between the RNA-seq datasets resulting from the compounds. Selected genes for the clustering were all downregulated by QN-302 in MIA-PaCa2 cells with the criteria Log_2_FC <  − 0.5 and false discovery rate (FDR) < 0.05. For the other datasets, any differentially expressed genes (DEGs) that did not pass FDR < 0.05 criterion was set to 0 i.e. the clustering was performed with statistically significant DEGs.

**Figure 3:**
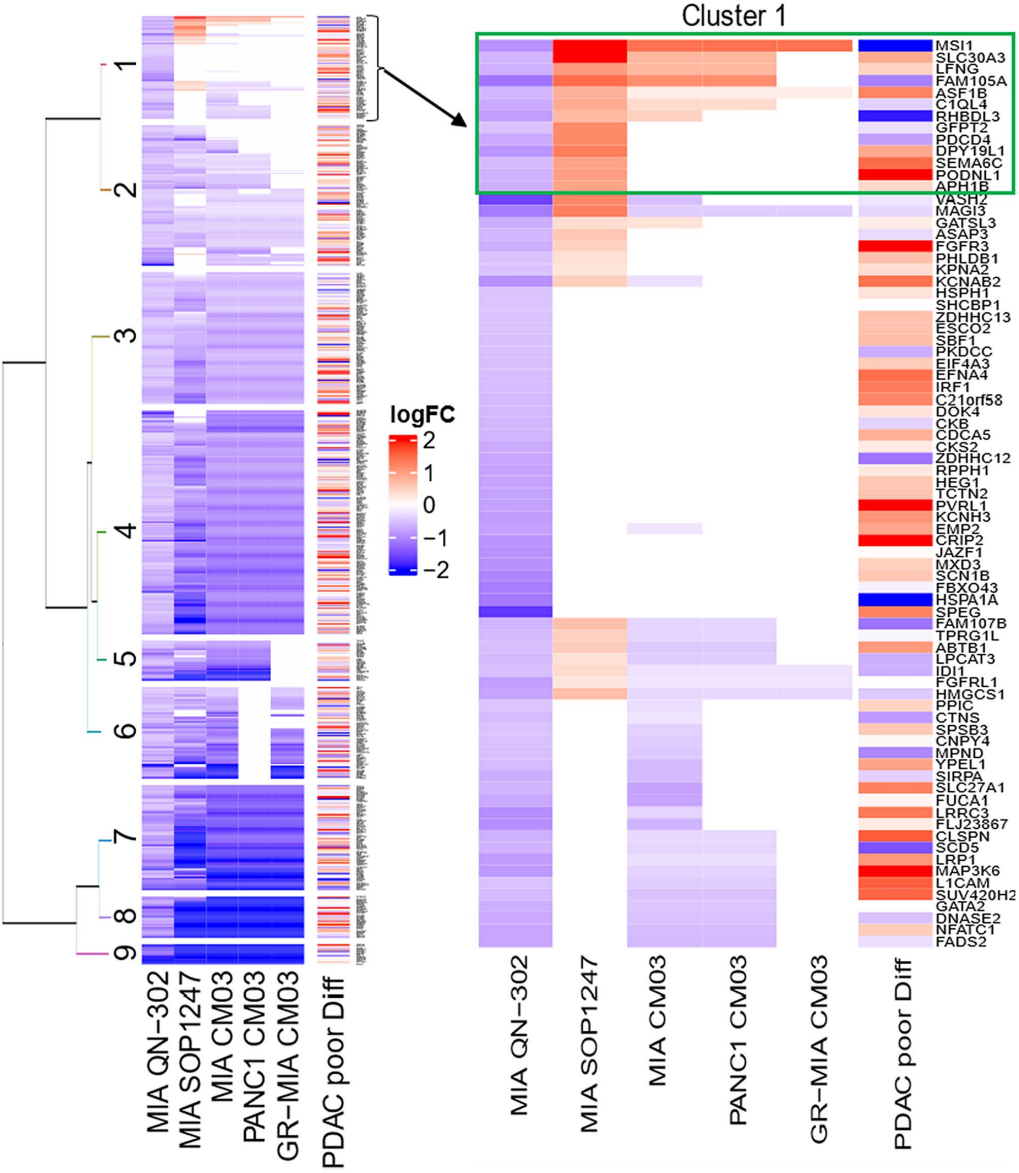
Heat-map showing the nine clusters of differentially regulated genes in MIA-PaCa2 and PANC-1 cell lines following drug treatment. Dark-blue strips indicate the most down-regulated genes and bright red those that are the most up-regulated, in terms of log_2_FC values. Genes in cluster one are solely down regulated by QN-302 treatment (see also the sub-set enclosed in the box, which are detailed in Table 3). The right-hand column indicates the status of genes in a small cohort of poorly differentiated human PDAC tumour tissues, as previously reported^44^. The moderate correspondence with the QN-302 gene cluster is likely to reflect the small PDAC tumour sample size.

The clustering resulted in nine clusters (Fig. 3). Cluster one is specific to genes down-regulated solely by QN-302 while other clusters contain shared down-regulated genes between all or some of the datasets e.g. clusters seven, eight and nine contain strongly downregulated genes in all datasets but cluster six is shared by all apart from the CM03-PANC1 dataset (Fig. 3).

Cluster one contains 77 QN-302-specific down-regulated genes with some also down-regulated in one or two of the other datasets. Therefore, additional filters were applied to pull out highly QN-302-specific subsets of genes by removing any gene that is downregulated (Log_2_FC < 0) in either one of the other datasets (Table 1). Table 3 lists the most significant members of this group. All have smaller log_2_FC changes than the genes highlighted in Table 2, although several such as *GFPT2*, *ASF1B* and *MSI1*, have previously been reported as having a role in PDAC (see below). The first two have elevated expression in several human tumor types (https://www.proteinatlas.org).

**Table 3:** Log_2_FC values for those genes (in cluster 1 of Fig. 3) selectively down-regulated by QN-302 and not by the other derivatives, showing Log_2_FC values across all datasets.

Gene	PQS	QN-302 in MIA-PaCa2	SOP-1247 In MIA-PaCa2	CM03 in MIA-PaCa2-GemR	CM03 in PANC-1	CM03 in MIA-PaCa2	Role in cancer
PODNL1	12	− 0.67	0.99	0.14	1.04	0.22	Promotes cell proliferation and migration in glioma via regulating Akt/mTOR pathway
SEMA6C	11	− 0.60	0.96	0.02	0.71	1.12	Suppresses proliferation of PDAC via Inhibition of AKT/GSK3/β-catenin, cyclin D1 pathways
ASF1B	6	− 0.62	0.81	0.19	0.18	1.02	Promotes malignancy and EMT process in PDAC cells. High expression in PDAC
SLC30A3	5	− 0.65	2.47	0.75	2.33	1.02	Tumour suppressor in glioblastoma
LFNG	30	− 0.67	1.04	0.75	0.80	0.29	Tumour suppressor in PDAC
APH1B	1	− 0.68	0.94	0.23	0.10	0.26	Part of Notch signalling modulating
GFPT2	17	− 0.52	1.18	0.28	1.49	1.92	Overexpressed in hypoxic PDAC
FAM105A	7	− 1.18	1.46	1.15	1.41	1.14	Unknown
MSI1	29	− 0.94	2.47	1.41	2.11	2.40	Notch signalling as a central regulator of PDAC

All listed genes in the QN-302 dosed cell line have *P* values < 0.05.

### Molecular modelling and simulations

**Figure 4:**
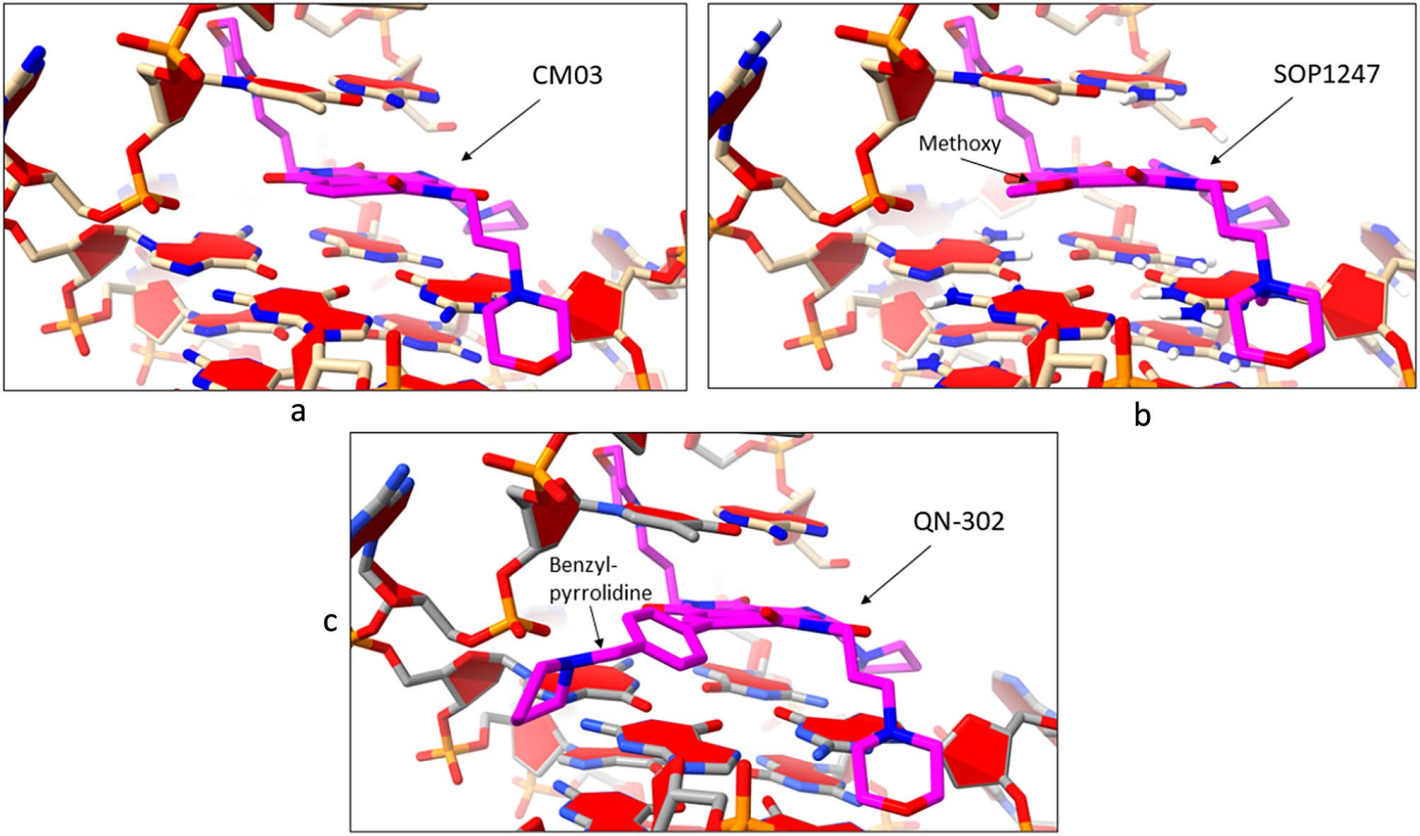
(**a**–**c**) Stick views of the putative ligand binding site at the junction of a duplex-G4 complex^61^, in each case with a bound ligand, having its carbon atoms coloured mauve. The ligand positions are based on those previously determined for QN-302^44^. In Fig. 3b the methoxy substituent of SOP1247 is in an energetically favourable conformation. In Fig. 3c the phenyl ring of the benzyl-pyrrolidine substituent of QN-302 is stacked over a guanine ring of the neighbouring G-quartet.

**Figure 5:**
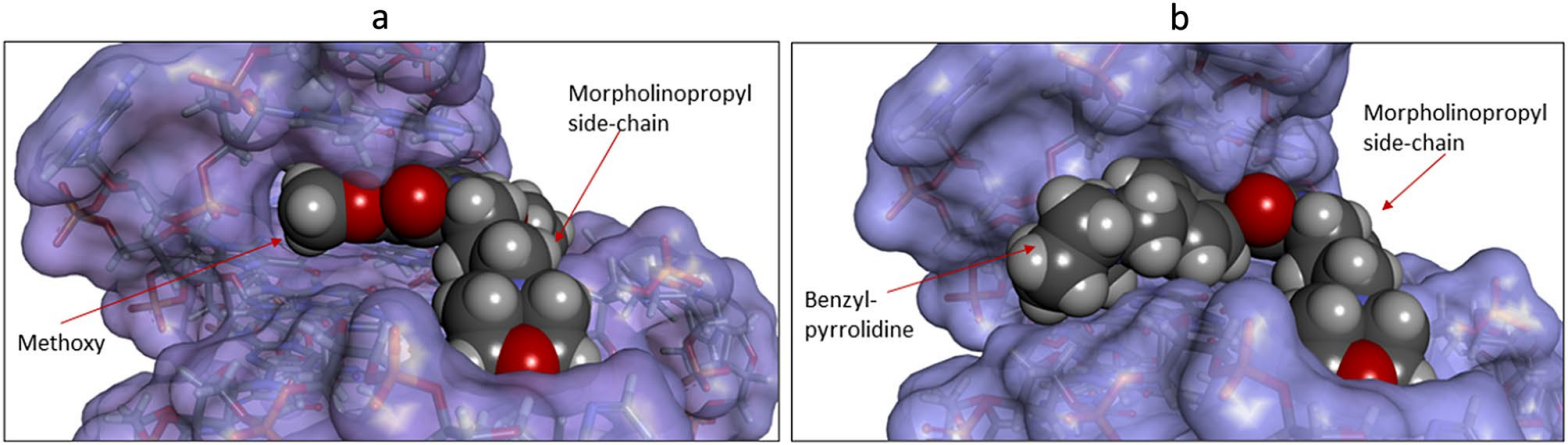
(**a**) A view of the SOP1247 G4-duplex structure, with the G4-duplex shown in solvent-accessible surface representation and the ligand in space-filling mode, coloured according to atom type. The methoxy group is shown fitting snugly into a cavity of the groove. (**b**) The same view with now the QN-302 molecule bound. The benzyl-pyrrolidine substituent is seen to extend deep into the groove, contacting one side of the groove surface. The remaining space in the groove may contain a cluster of conserved structured solvent molecules, as observed experimentally in several earlier co-crystal structures of G4-naphthalenediimide complexes^63^.

**Figure 6:**
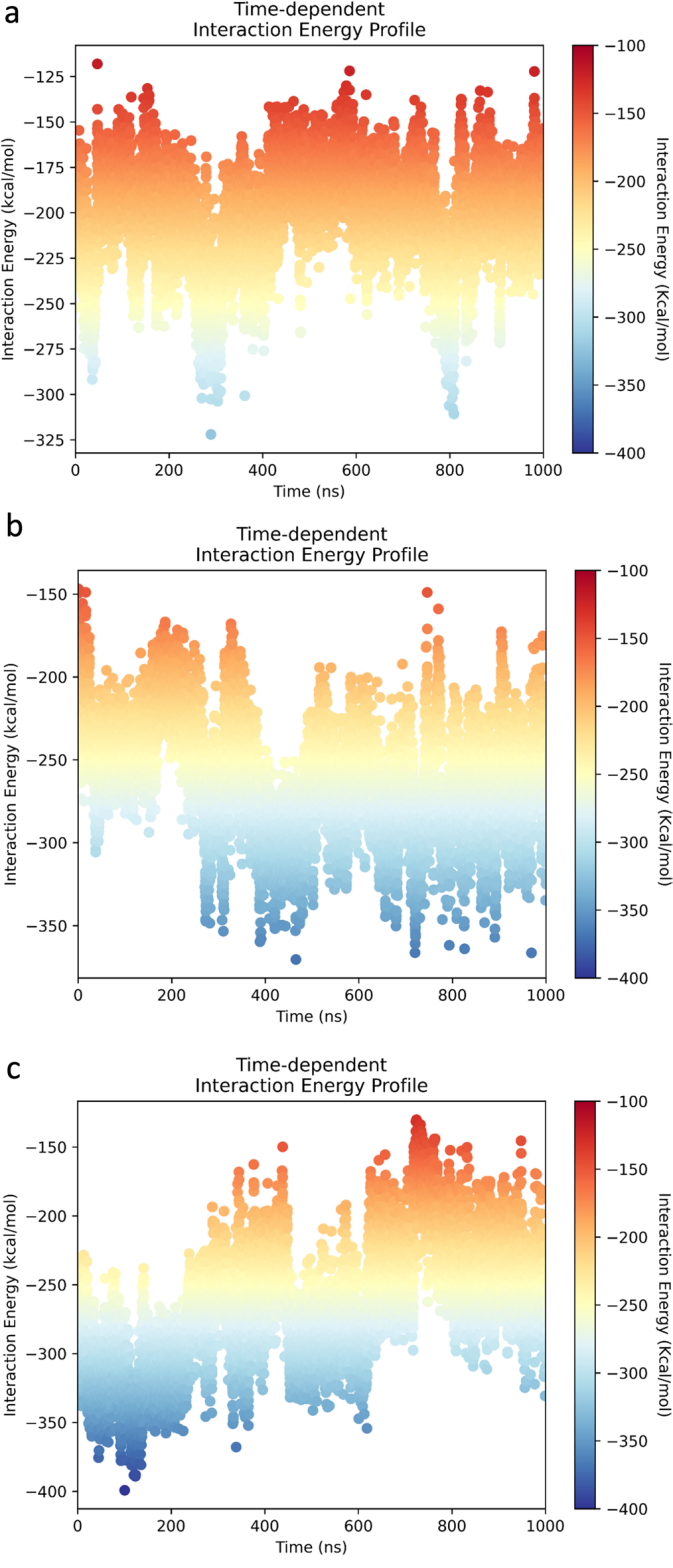
The interaction energies of (**a**) CM03, (**b**) SOP1247 and (**c**) QN303, as calculated from the 1000 ns molecular dynamics simulations. An extended region of higher binding energy for QN-302 is apparent for much of the simulation.

The modelling studies suggest that compound CM03 binds at the duplex-G4 junction of the G4 with its three pendant sidechains each residing in a groove of the G4 and the naphthalene diimide chromophore stacking onto the terminal G-quartet of the G4 (Fig. 4a), in accord with earlier molecular modelling of CM03 bound to a parallel human telomeric G4^40^. Compound SOP1247 has its fourth substituent, a methoxy group, protruding into the groove (Figs. 4b, 5a). The longer benzyl-pyrrolidine substituent of QN-302, by contrast, protrudes significantly further into the groove (Fig. 5b) and its phenyl ring can effectively stack onto the adjacent guanine of the lower G-quartet (Fig. 4c). The ΔG values for the docked poses for CM03, QN302 and SOP1247 are − 171.9, − 263.7 and − 199.2 kcal/mol respectively. The molecular dynamics simulations reveal that all the complexes were stable and the side chains of the ligand are able to effectively interact with DNA grooves at the G-quadruplex-duplex junction. The average binding energies were calculated to be − 201.19, − 265.98 and − 261.35 for CM03, QN302 and SOP1247 respectively (Fig. 6).

## Discussion

This study has shown that comparing detailed transcriptomic data from RNA-seq analyses has revealed significant differences in the patterns of down-regulated genes for the most potent compound QN-302 compared to the two closely related compounds CM03 and SOP1247. QN-302 is significantly more potent than the two analogues in a panel of pancreatic cancer cell lines. Dynamics simulations as well as experimental data show that QN-302 stabilizes a representative G4 structure to a greater extent than the other two. This suggests that the methoxy addition to compound CM03, resulting in compound SOP1247, has only minor effects on G4 binding, cell growth inhibition (Table 1) or the profile of downregulated genes (Table 2). In striking contrast, the benzyl-pyrrolidine substituent on QN-302 has resulted in enhanced G4 binding, probably due in large part to the phenyl group stacking onto the adjacent G-quartet, as suggested by molecular modelling^44^. It also protrudes further into the G4 groove, filling much of the available space (Fig. 5b). It is plausible to speculate that this large pendant group would also enhance QN-302 affinity for some G4s that have a comparable groove in an equivalent accessible position, while also enhancing selectivity, although this remains to be demonstrated. We conclude that the benzyl-pyrrolidine group is responsible for enhanced cellular and in vivo potency, enhanced G4 binding and greater selectivity in the pattern of downregulated gene expression that it produces, at least in the MIA-PaCa2 cell line. QN-302 is more hydrophobic than SOP1247 or CM03, which may contribute to its superior cellular potency. Its half-life (T_1/2_) in vivo of 37 h^42^ is comparable to that of CM03 (33 h) and SOP1247 (32 h: unpublished data). Values for bioavailability as defined by AUC_all_ (in ng.hr/mL) for CM03, QN-302^42^, and SOP1247 are 11,113, 5863 and 10,876, suggest that pharmacokinetic issues alone are not responsible for the superior activity in vivo of QN-302.

QN-302 mostly affects the same pathways as CM03 and SOP1247, but often has a greater effect on genes distinct from those sensitive to CM03 and SOP1247, in any one individual pathway (Table 2). Thus QN-302 downregulates expression of the *GLI1* gene encoding the major transcription factor GLI1 in the Hedgehog pathway to a greater extent than the other two compounds, which mostly affect expression of the *GLI4* gene (Table 4). The GLI1 protein is significantly upregulated in human PDAC^45–47^ and thus has been considered a potential therapeutic target^48^, since its up-regulation promotes migration and metastasis^46^. Genes for several other components of the Hedgehog pathway^49^ are also downregulated by QN-302 (Table 2). The down regulation of other QN-302-specific genes (Table 3), although individually at modest levels, may also, we suggest, contribute cumulatively to the drug’s anticancer activity in view of their role in PDAC tumorigenesis. The *ASF1B* gene, upregulated in PDAC, codes for histone chaperone 1B and is involved in PDAC progression^50,51^ by activating c-MYC^52^. The *GFPT2* gene, also up regulated in human PDAC^53^, encodes for glutamine‑fructose‑6‑phosphate transaminase 2, a key enzyme in the hexosamine biosynthesis pathway.

**Table 4:** Log_2_FC values for genes in the Hedgehog pathway down regulated by QN-302, for 24 h exposure, together with numbers of Putative G-quadruplex Sequences (PQS), from Ref.^42^. CM03-R is the gemcitabine-resistant MIA-PaCa2 and CM03-PANC is the PANC-1 cell line.

Gene	QN-302 in MIA-PaCa2	SOP1247 in MIA-PaCa2	CM03 in MIA-PaCa2	CM03 in MIA-PaCa2-GemR	CM03 in PANC-1	PQS
KIF7	− 0.62	− 1.46	− 1.32	− 1.34	− 0.67	13
SMO	− 0.86	− 1.42	− 1.21	− 0.33*	− 0.82	12
SUFU	− 0.22	− 0.16	− 0.26	− 0.14*	0.12*	20
GLI1	− 1.86	− 0.13	− 0.64	− 0.59*	− 0.57	15

All *P* values are < 0.05, apart from those marked*.

We suggest that the enhanced downregulation of several genes known to be significant in PDAC (Tables 2, 3), may be a consequence of selectivity at the G4 level. However, at present this must remain speculative in the absence of the identification of the target G4(s) within each gene, as well as detailed binding affinity data on the G4s. Many of these genes are G4-rich (Table 2). However, it is yet to be established as to which ones form stable G4s and play a significant role in transcriptional downregulation induced by QN-302. We have recently identified a plausible G4 sequence in the promoter of the *S100P* gene, 48 nucleotides upstream from the transcription start site^44^, which forms a stable G4 structure under physiological K^+^ conditions, and which is further stabilized by QN-302. *S100P* has frequently been identified as an upregulated gene in PDAC^48,53–59^ and both the gene and the S100P protein are plausible therapeutic targets in PDAC, with cancer cell apoptosis and anti-tumor activity being consequences of targeting^57,58^.

The present study is unable to fully answer the question of which genes are responsible for the high potency of QN-302. We suggest that at least some of the genes highlighted here (Tables 2, 3, Cluster1) are centrally involved, not least those that are selective for the drug, such as *GLI1*, *S100P*, *CLIC3* and *NTN4*. Previous studies have demonstrated that S100P^57,60^ is a viable target in human cancers including PDAC, probably by inducing p53-related apoptosis^58^. The interaction of the GLI1 protein with selective quinoline-based small molecules^47^ has been shown to down-regulate the Hedgehog signaling pathway by inhibiting GLI1-DNA binding and hence the transcription of GLI1 target proteins. This resulted in dose-dependent apoptosis in cancer cells, although the details of the mechanism involved have yet to be disclosed. This study has also shown that a decrease of *GLI1* expression in a dose-dependent manner in an in vivo xenograft model for melanoma suggested the use of *GLI1* as a mechanistic biomarker of response. Knock-down of GLI1 expression leads to apoptosis via downregulation of BCL-2 and BCL-xl expression^61^. QN-302 has been shown to induce apoptosis in vivo^42^, consistent with these other studies.

## Materials and methods

### G4 ligands

Syntheses of compounds CM03 and QN-302 have been previously described^40,42^. All compounds were used for biophysical and biological studies as their 99% pure free bases.

### Synthesis of SOP1247

SOP1247 was synthesized and purified using the analogous procedure to QN-302, with a methoxy substitution on the naphthalene diimide core instead of the phenyl-substituted 2-(pyrrolidin-1-yl)ethyl)amino group in QN-302 (Fig. 1).

NaOMe (0.5 M in THF) (843 µl, 0.422 mmol) was added to a solution of 4-bromo-2,7-bis(3-morpholinopropyl)-9-((2-(pyrrolidin-1-yl)ethyl)amino) benzo[lmn][3,8] phenanthroline-1,3,6,8(2H,7H)-tetraone (100 mg, 0.141 mmol) and copper(I) iodide (53.5 mg, 0.281 mmol) under N_2_ in THF (1 mL) and stirred at 60 °C for 2 h. The reaction was partitioned between water (10 mL) and DCM (10 mL) and the layers separated via a hydrophobic frit. The aqueous layer was extracted with DCM (2 × 2 mL) and the combined organic extractions concentrated *in vacuo*. The crude product was purified by chromatography on silica gel (12 g column, 0–8% (0.7 M Ammonia/MeOH)/DCM, liquid load in DCM) to afford 4-methoxy-2,7-bis(3-morpholinopropyl)-9-((2-(pyrrolidin-1-yl)ethyl)amino)benzo[lmn][3,8]phenanthroline-1,3,6,8(2H,7H)-tetraone (31 mg, 0.043 mmol, 31% yield) as a dark fuchsia solid.

^1^H NMR in CDCl_3_ 1871-29-P1 was consistent with product structure at 92% purity. ^1^H NMR (400 MHz, Chloroform-d) δ 9.99 (t, J = 5.4 Hz, 1H), 8.39 (s, 1H), 8.30 (s, 1H), 4.35–4.24 (m, 4H), 4.23 (s, 3H), 3.80 (s, 2H), 3.71–3.58 (m, 8H), 3.01 (t, J = 6.4 Hz, 2H), 2.79 (s, 4H), 2.55 (td, J = 6.9, 2.7 Hz, 4H), 2.49 (s, 8H), 2.03–1.88 (m, 8H). Contains ca. 8 mol% of the starting bromide. UPLC, Basic, 1871-29-P1, m/z 663.1 [M^+^H]^+^ (ES^+^); at 1.38 min, 99% purity @ 210–400 nm.

NMR and mass spectra are available in the Supplementary Data. The mass spectral peak at 663.648 Da corresponds to the molecular weight of the unfragmented pure compound.

### SRB assays

Cell lines (MIA-PaCa2, PANC-1, BxPC-3 and Capan-1) were purchased from ATCC (cat #: CRL-1420, CRL-1469, CRL-1687 and HTB-79). The former two cell lines were maintained in DMEM and the latter two cell lines in RPMI-1640 and IMEM, respectively. All media were supplemented with 10% foetal bovine serum (FBS) (ThermoFisher, cat #: 10270106), 2 mM L-glutamine (Sigma-Aldrich, cat #: D6429), 0.1 mg/ml streptomycin and 100 U/ml penicillin (Sigma-Aldrich, cat #: P4333). Specifically, MIA-PaCa2 medium was also supplemented with 2.5% horse serum (ThermoFisher, cat #: 16050130) and Capan-1 medium with extra 10% FBS to make 20% in total. The gemcitabine resistant MIA-PaCa2 cells were generated by incremental increases of gemcitabine concentration and were maintained in the same culture conditions as parental MIA-PaCa2. Cell lines were maintained at 37 °C, 5% CO_2_ and passaged or their media were changed every 2–3 days. The cell lines were routinely tested to ensure that they were mycoplasma-free by an RT-qPCR-based method. Briefly, cells were seeded at appropriate densities into the wells of 96-well plates in their corresponding medium and incubated overnight to allow the cells to attach. Subsequently cells were exposed to freshly made solutions of drugs and incubated for a further 96 h. Drugs were dissolved in H_2_O, with the judicious addition of a few drops of 0.1 M HCL to facilitate solubilization, and then filtered through 0.22 µm pore-size filter units (stock 10 mM) before addition to appropriate cell line media in quadruplicate at a range of final concentrations. Cellular growth inhibition was measured using the sulforhodamine B (SRB) assay in 96 well plates as described previously^37^. 50% inhibitory concentrations (GI_50_) were determined by taking the mean absorbance at 540 nm for each drug concentration expressed as a percentage of the absorbance of untreated control wells.

### G4 melting assays

FRET DNA melting assays on CM03, SOP1247 and QN-302 were performed using a fluorescence resonance energy transfer (FRET) assay^43^. The labelled oligonucleotide had the fluorophores FAM (6-carboxyfluorescein) as donor and fluorophore TAMRA (6-carboxytetramethyl-rhodamine) attached as acceptor. The sequence used was:

Human telomeric G4: 5′-FAM-d(GGG[TTAGGG]_3_)-TAMRA-3′

The detailed protocol was as previously described^38^, with a 60 mM potassium cacodylate buffer (pH 7.4).

### RNA-seq analysis of RNA from cell-based studies

The methodology used has been previously reported in detail^40^ and the process of determining changes in transcription on exposure of PDAC cells to the compounds has been fully described. The RNA-seq data sets are available in the GEO public functional genomics data repository (https://www.ncbi.nlm.nih.gov/geo/), as

- GSE151741 for QN-302
- GSE234779 for SOP1247
- GSE105083 for CM03 in MIA-PaCa2 cells
- GSE148200 for CM03 in gemcitabine-resistant MIA-PaCa2 cells
- GSE105083 for CM03 in PANC-1 cells

RNA-seq analyses have been previously reported^40–42^ for all the above apart for compound SOP1247. The experimental and analytical protocols used in this instance were identical to those used for the other compounds.

### Bioinformatics analysis

Prior to clustering analysis, the gene expression data files were filtered to include only genes that met specific significance criteria, using purpose-written PYTHON scripts. Genes with log_2_ FC <  − 0.5 or > 0.5 and a FDR < 0.05 to eliminate any that are unlikely to be statistically significant, were retained for further analysis. For G4-drug datasets, downregulation patterns were emphasized on the basis that these were the consequence of G4 promoter targeting. In the hierarchical clustering analysis, we focused on downregulated genes specifically in QN-302 (log_2_FC <  − 0.5 and FDR < 0.05). Log_2_FC values were set to 0, if they were not significant (FDR < 0.05) to focus clustering on statistically significant DEGs only. Hierarchical clustering was performed using Ward's linkage method with the Manhattan distance protocol (the sum of the right-left clustering plus the sum of all the up-down clustering) as the similarity metric. The hierarchical clustering was implemented using the ComplexHeatmap package^62^, version 2.14.0 R (https://bioconductor.org/packages/release/bioc/html/ComplexHeatmap.html). After hierarchical clustering was completed, the resulting dendrogram was analyzed to identify distinct clusters of genes based on their expression patterns. We started by cutting the dendrogram tree into nine clusters.

The number of putative G4 sequences (PQS) in an individual gene were estimated from the occurrence of the canonical G4 motif (G_≥3_N_1-7_G_≥3_N_1-7_G_≥3_N_1-7_G_≥3_). The same criterion was used for gene promoter sequences (defined as being up to 2 kilobases upstream of the transcription start site (TSS) and 100 bases downstream) and in exons and introns^40–42^.

### Molecular modelling and dynamics simulations

A molecular model for QN-302 docked into the junction of a parallel G-quadruplex-duplex crystal structure^63^, has been previously described^44^ and was used here. This employed the crystal structure of the parallel stranded quadruplex^64^ formed from the human telomeric sequence and its complex with the earlier-generation naphthalene diimide compound MM41^38^ (PDB entry 3UYH) as starting models to assess how QN-302 interacts with parallel topology quadruplexes in general^65^. The docking used MolSoft ICM 3.9-3a software (https://www.molsoft.com/). The structures of compounds CM03 and SOP1247 were generated in the low-energy position found for QN-302. No potentially disruptive non-bonded interactions were apparent. Analyses and visualizations were undertaken with the program ChimeraX (https://www.cgl.ucsf.edu/chimerax/). The docked complexes were then subjected to molecular dynamics simulations in explicit solvent. The ligands were parameterized using the GAFF forcefield^66^, while the nucleic acids were described using the ParmBsc1 force field with OL15 modifications^67^. The two K+ ions in the G-quadruplex were treated as a part of the structure. Each system was solvated in a cubic box containing TIP3P water molecules^68^ and 100 mM KCl^69^. The complexes were minimized for 3000 steps of steepest descent and equilibrated for 5 ns before the production run. During the equilibration step, each system was slowly heated to 300 K in an NPT ensemble. The Langevin thermostat and Berendsen barostat were used to stabilize the temperature and pressure respectively. The final production run was set to 1000 ns in the NVT ensemble. All the simulation protocols were identical and were run using the ACEMD v3.5 MD engine^70^. The interaction free energy between the ligand and the DNA was calculated using the NaMD energy plugin implemented in the VMD package^71^.

### Supplementary Information

Supplementary Information.

## Data Availability

All transcriptome data sets have been deposited and are publicly available from the GEO public functional genomics data repository (https://www.ncbi.nlm.nih.gov/geo/).
