# Supplementary material for: Structure–activity relationships for the G-quadruplex-targeting experimental drug QN-302 and two analogues probed with comparative transcriptome profiling and molecular modeling
Source: Sci Rep. 2024 Feb 11;14:3447. doi: 10.1038/s41598-024-54080-2 (PMC10859377; doi:10.1038/s41598-024-54080-2)

SUPPLEMENTARY DATA

Structure-activity relationships for the G-quadruplex-targeting experimental drug QN-302 and two analogues probed with comparative transcriptome profiling and molecular modeling

Ahmed Abdullah Ahmed^1,2^, Maria Roman-Escorza^2^, Shuang Chen^1^, Richard Angell^1,3^, Sally Oxenford^1,4^, Matthew McConville^5^, Naomi Barton^6^, Mihiro Sunose^5^, Dan Neidle^6^, Shozeb Haider^1^, Tariq Arshad^7^ and Stephen Neidle^1*^

^1^The School of Pharmacy, University College London, London WC1N 1AX, UK

^2^Now at Guy’s Cancer Centre, Guy’s Hospital, London SE1 9RT, UK

^3^Now at Medicines Discovery Institute, Cardiff University, Cardiff, CF10 3AT, UK

^4^Now at Artios Ltd, Cambridge, CB22 3FH, UK

^5^Sygnature Discovery Ltd, BioCity, Nottingham NG1 1GR, UK

^6^Tax Policy Associates, London EC1R 0ET, UK

^7^Qualigen Therapeutics Inc, Carlsbad, CA 92011, USA

1. NMR and (b) mass spectral data for SOP1247

(a)


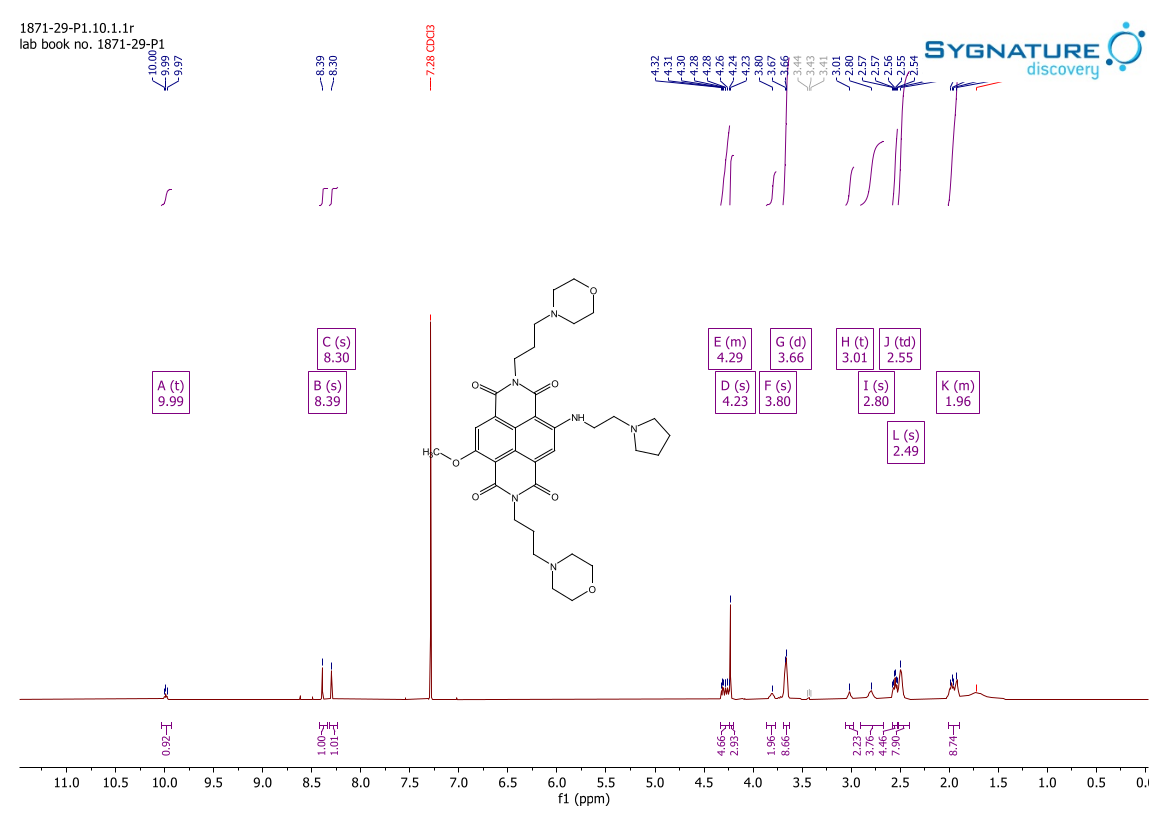


(b)
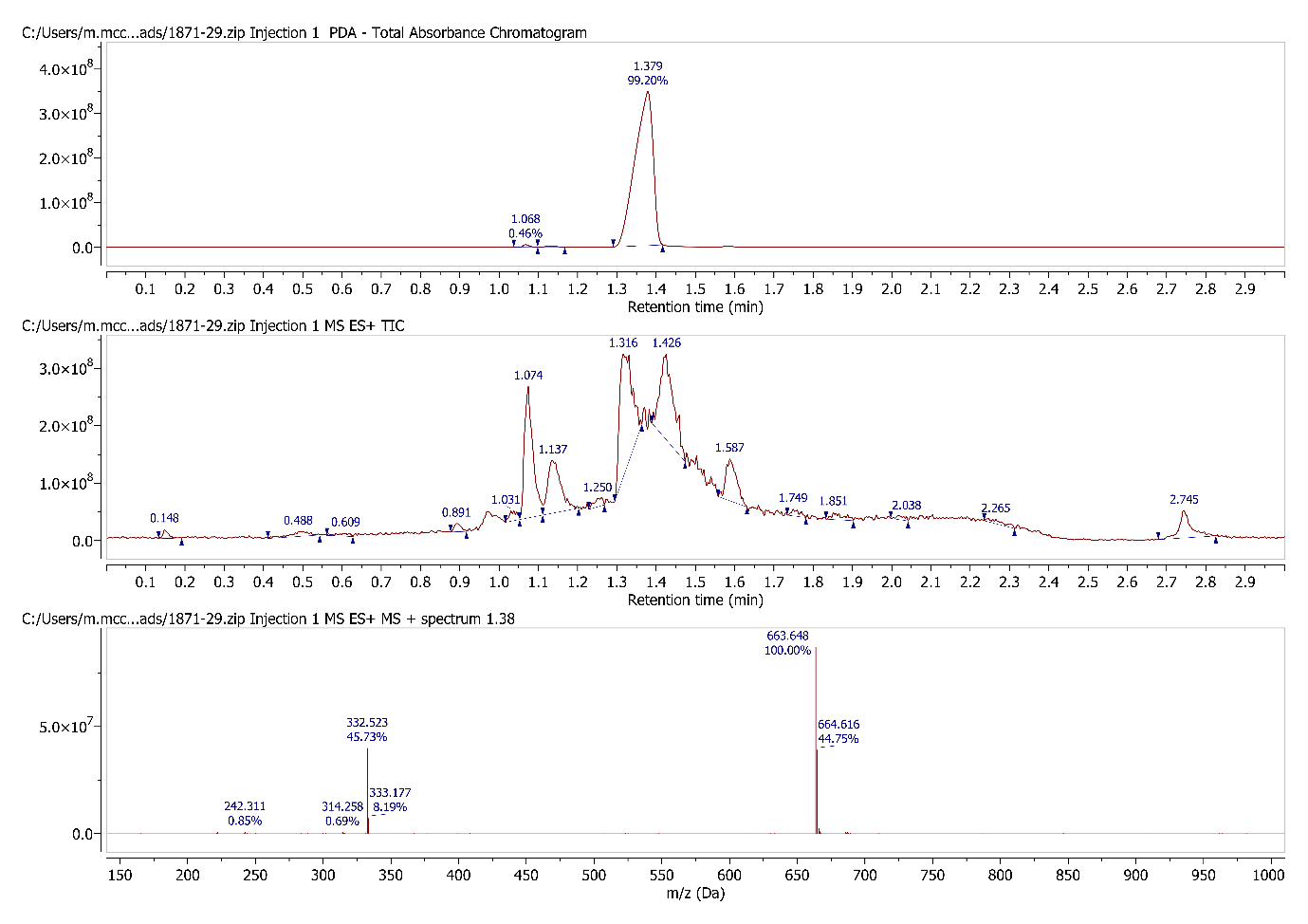

Supplement: Supplementary file 1 — Supplementary Information. [file 41598_2024_54080_MOESM1_ESM.docx]
